# Book Reviews

**Published:** 1889-04

**Authors:** 


### BOOK REVIEWS.

Handbook of the Diagnosis and Treatment of Diseases of the Throat, Nose and Naso-Pharynx. By Carl Seiler, M. D. Third edition, 12mo. Pp. xii., 373. Philadelphia: Lea Brothers & Co. 1889. Chicago: A. C. McClurg & Co.

Physicians who are familiar with the manual on Microscopical Technology by this author need not be told that this handbook is a good one. Many of the good qualities of the former are to be found in the latter. Few medical writers surpass this author in ability to make his meaning perfectly clear in few words, and in discrimination in selection, both of topics and of methods. In this handbook the methods described are his own, but whenever his instruments or ideas are borrowed he mentions their source, and gives ample credit to their originators. In this connection it is pertinent to observe that when he makes an alteration in an instrument he has a better reason than a simply vainglorious desire to attach his own name, along with the new device, to the instrument. A case in point is his attachment to the Jarvis snare, to be used in removing anterior nasal hypertrophies.

In addition to the points mentioned, what may be called the distinguishing features of the book are as follows: 1. A chapter on the physiology of the voice and articulate speech.

2. A blank form for records of cases, the use of which will facilitate taking a record of conditions and also insure thoroughness in observing and recording them.

3. The illustrations, many of which are new and quite unusually well drawn.

4. A chapter on hay-fever. The author, in common with many others, believes that the disease may be controlled, but he does not exactly agree with his illustrious fellow townsman, the editor of the Annual of the Universal Medical Sciences, as to the comparative value of the galvano-cautery, and of acids in the treatment of the lesion.

The author evidently never has himself been a sufferer with that form of chronic pharyngitis known as *p. sicca* or he would not dismiss it in so summary a way as he does.

The chromolithographic plates which are bound with the text illustrate well the imperfections and apparent limitations of that art.

The book deserves a large sale, especially among general practitioners, hackneyed though the phrase is. E. W.

Surgical Bacteriology. By Nicholas Senn, M.D., Ph.D., Professor of Principles of Surgery and Surgical Pathology, Rush Medical College, Chicago. 8vo., pp. 270. Philadelphia: Lea Brothers & Company. 1889. Chicago: W. T. Keener.

The twenty-two chapters of this book are devoted to the following subjects: Hereditary transmission of microbic diseases; Do pathogenic microörganisms exist in the healthy body?; Sources of infection; Localization of microbes; Elimination of pathogenic microörganisms; Antagonism amongst microörganisms; Inflammation; Suppuration; Gangrene; Septicæmia; Pyæmia; Erysipelas; Erysipeloid; Noma; Tetanus; Tuberculosis; Anthrax; Glanders (Malleus Humidus); Actinomycosis hominis; Gonorrhoea; Syphilis; On the alleged microbic origin of tumors. Each one of these subjects is discussed with the care, minuteness, and exactness characteristic of all Dr. Senn's work. They that recognize the fact that surgical pathology is now almost synonymous with surgical bacteriology should not fail to study the book; others should study it and become convinced. It contains some well executed colored plates, reproduced from *Klebs' Lehrbuch der pathologischen Anatomie*.

Intestinal Surgery. By N. Senn, M. D., Ph. D. 8vo. Pp. vii and 269. Price $2.50. Chicago: W. T. Keener. 1889.

The limits of a book notice will not permit an adequate review of this volume, and this fact may excuse a quotation from the preface: "While the following pages are not intended to serve the purpose of a complete text-book on Intestinal Surgery, still the author hopes that they may contain some new facts and suggestions which will prove useful to those who practice this branch of surgery." "The first part of the book contains a résumé of the best literature on the subject of intestinal obstruction which has been arranged in a systematic manner for ready reference." "The second part represents the author's own original work, made with special reference to the treatment of intestinal obstruction and the diagnosis of perforation of the intestinal canal; to which is added the report of three cases of gunshot wound of the abdomen, in which inflation with hydrogen gas proved a positive test in making a correct diagnosis, before the abdomen was opened. One of the principal objects in publishing these papers in book form is to stimulate the young men in our profession to enter the field of original investigation, as the author is firmly convinced that experimental research constitutes the shortest and safest route to the perfection of the principles and practice of intestinal surgery."

Those who are familiar with the author's style of work know what this volume must be, and those who are not, may be assured that it is eminently creditable to the author himself, and an honor to the cisatlantic branch of the medical profession.

THE INSANE IN FOREIGN COUNTRIES. By WILLIAM P. LETCHWORTH, President of the New York State Board of Charities. 8vo., pp. xii, 374. New York and London: G. P. Putnam's Sons. 1889.

It is a positive pleasure to notice this work. It is the outcome of an investigation of foreign charitable institutions, pursued without interruption for seven months, in which time special attention was given to the various kinds of provision made for the insane poor. Stenographic notes of visitations and interviews were made, and thus valuable opinions, expressed by distinguished specialists, with whom the treatment of the mentally diseased has been a life-long study, have been carefully noted. Mr. Letchworth has avoided medical theories and technicalities, and has presented the subject in plain language. Of the publishers' part, it is sufficient to say that the book comes from the Knickerbocker press, and is in that tasteful style for which the publishers are noted. It is well illustrated.

CLINICAL LECTURES ON DISEASES OF THE URINARY ORGANS, delivered at University College Hospital. By SIR HENRY THOMPSON. Eighth Edition. 8vo., pp. xiv., 470. Philadelphia: P. Blakiston, Son & Co. 1888. Chicago: W. T. Keener.

The first edition of this book, published twenty years ago, contained twelve lectures, the seventh edition twenty-six, and the present thirty-two. In the present edition the chief additions relate to the supra-pubic operation for stone and for tumor, to the results of digital exploration of the bladder, to the recent modes of affording relief by operation in cases of advanced prostatic disease, to the latest operative treatment for tumors of the bladder, and embraces a *résumé* of the author's entire experience of operations for calculus, made up to the year 1886, numbering about 900 cases. All the material that was given in former editions has been thoroughly rewritten. The work has been translated into French, German, Italian, Spanish and Russian.

HAND-BOOK OF PHYSIOLOGY. By W. MORANT BAKER, F.R.C.S., late Lecturer on Physiology at St. Bartholomew's Hospital, etc., and VINCENT DORMER HARRIS, M.D., Demonstrator of Physiology at St. Bartholomew's Hospital, etc. Twelfth Edition, rearranged, revised and rewritten, with 500 illustrations. 8vo., pp. xi, 784. New York: William Wood & Company. 1889. Chicago: W. T. Keener.

This is the twelfth edition of the well-known Kirkes' Physiology, and contains a large amount of new matter, especially in the sections on the blood, the heart, and the muscular system, while the chapters on the nervous system, the reproductive organs, and on development have been rearranged, and to a great extent rewritten. Thus this popular work has been brought fully up to the present status of physiology. A work so well-known as this needs no further comment at our hands, especially when edited by such men as Mr. Baker and Dr. Harris.

THE OPERATIONS OF SURGERY. A Systematic Handbook for Practitioners, Students and Hospital Surgeons. By W. H. A. JACOBSON, F.R.C.S., Assistant Surgeon to Guy's Hospital, etc. With 199 illustrations. 8vo., pp. 1006. Philadelphia: P. Blakiston, Son & Co. 1889. Chicago: W. T. Keener.

This work is very comprehensive in scope and full in detail. The operations are well described and discussed as a rule, and the book may be commended as a safe guide to operative surgery.

A HANDBOOK OF THERAPEUTICS. By SIDNEY RINGER, M. D. Twelfth Edition. Pp. xi and 524. New York: William Wood & Co. 1889. Chicago: W. T. Keener.

A treatise which is so well and so favorably known as the subject of this notice needs no praise. The author states that in the preparation of this edition he has carefully revised every portion of it, and added much information both on new drugs and old. The plan is unchanged and it remains a work on clinical therapeutics of high value.

